# Supplementary material for: Highly metastatic claudin-low mammary cancers can originate from luminal epithelial cells
Source: Nat Commun. 2021 Jun 18;12:3742. doi: 10.1038/s41467-021-23957-5 (PMC8213728; doi:10.1038/s41467-021-23957-5)
Supplement: Supplementary file 1 — Supplementary Information [file 41467_2021_23957_MOESM1_ESM.pdf]

Supplementary Information

Highly metastatic claudin-low mammary cancers can originate from luminal epithelial cells

Patrick D. Rädler [1,2], Barbara L. Wehde [2], Aleata A. Triplett [2], Hridaya Shrestha [1], Jonathan Shepherd [3], Adam D. Pfefferle [3], Hallgeir Rui [4], Robert D. Cardiff [5], Charles M. Perou [3] and Kay-Uwe Wagner [1, *]

[1] Department of Oncology, Wayne State University School of Medicine and Tumor Biology Program, Barbara Ann Karmanos Cancer Institute, 4100 John R, EL01TM, Detroit, MI 48201, U.S.A.

[2] Eppley Institute for Research in Cancer and Allied Diseases, University of Nebraska Medical Center, 985950 Nebraska Medical Center, Omaha, NE 68198-5950, U.S.A.

[3] Department of Genetics, University of North Carolina at Chapel Hill, Lineberger Comprehensive Cancer Center, Chapel Hill, NC 27599, USA.

[4] Department of Pathology, Medical College of Wisconsin, Milwaukee, WI 53226, U.S.A.

[5] Center of Comparative Medicine, University of California, Davis, CA 95616, U.S.A.

* Lead Contact (correspondence to):

Dr. Kay-Uwe Wagner
Barbara Ann Karmanos Cancer Institute
4100 John R, mail code EL01TM
Detroit, MI 48201
U.S.A.

Tel: (313) 578-4334
Fax: (313) 578-4659
E-mail: wagnerk@karmanos.org

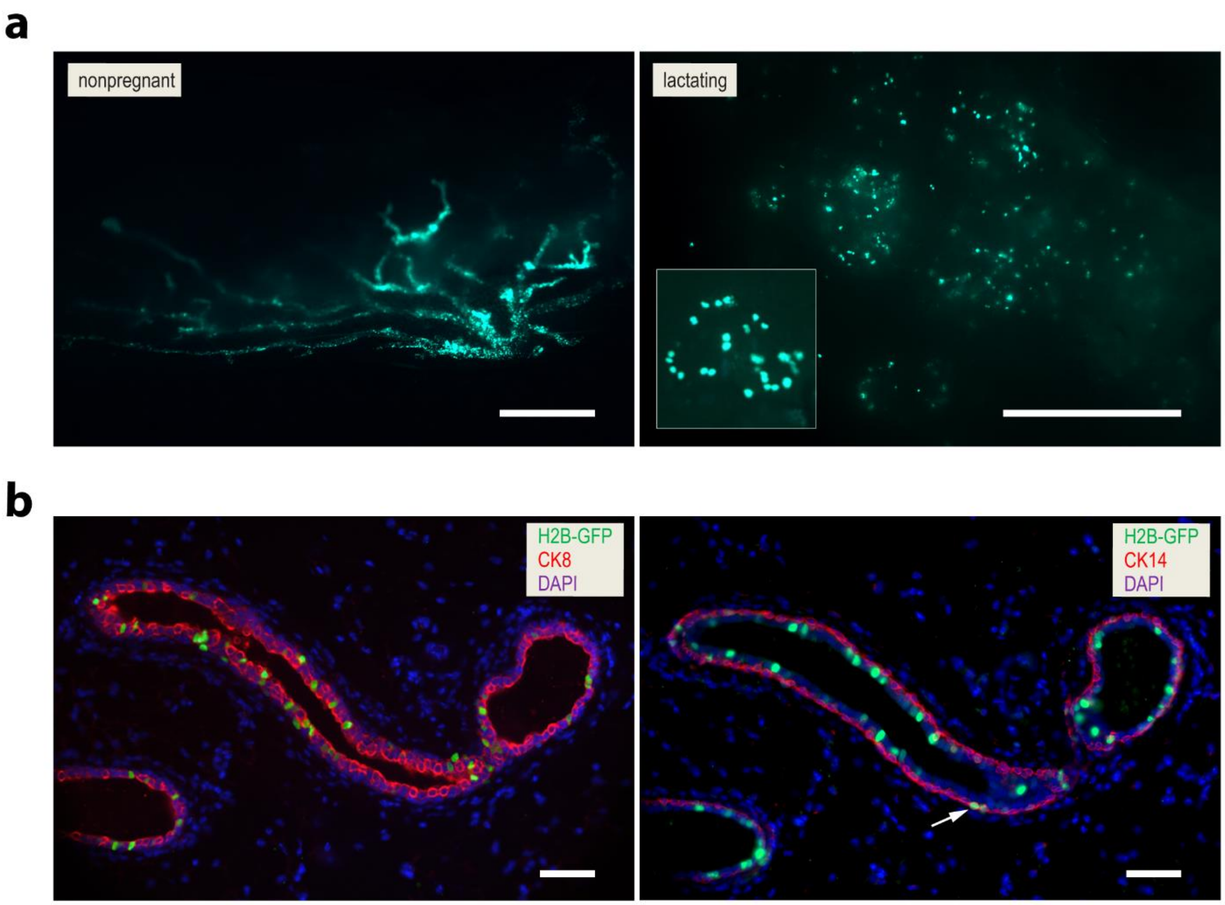

Supplementary Figure 1. The MMTV-tTA-mediated activation of the TetO-H2B-GFP reporter is largely confined to luminal epithelial cells

a. GFP fluorescent stereoscopic images of fresh mammary gland tissues from nonpregnant and lactating MMTV-tTA TetO-H2B-GFP double transgenic females (N = 3 biological replicates); bars, 1 mm.

b. Immunofluorescent staining of GFP in combination with the luminal Cytokeratin 8 (CK8) or basal Cytokeratin 14 (CK14) marker on serial mammary gland sections (N = 6 biological replicates) from a nonpregnant MMTV-tTA TetO-H2B-GFP female; bars, 50 µm. The arrow in the right panel points to a single CK14-positive basal epithelial cell expressing the nuclear H2B-GFP reporter.

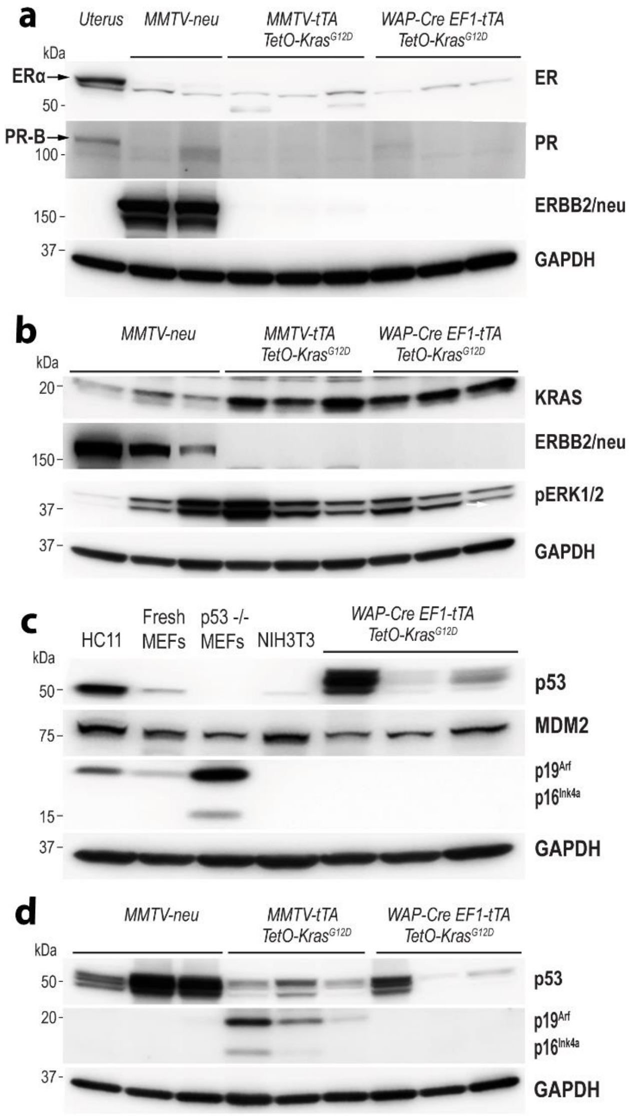

Supplementary Figure 2. Comparative analysis of KRAS expression, downstream activation of mitogen-activated protein (MAP) kinases, and Cdkn2/p53 pathway genes in mammary tumors that express oncogenic KRAS under control of the MMTV-LTR or constitutively active EF1 promoter

a. Immunoblot analysis of ERα, PR, and ERBB2 on mammary tumors that overexpress wildtype ERBB2/neu (MMTV-neu, N = 2 biological replicates) or oncogenic KRAS under the control of the MMTV-tTA (N = 3 biological replicates) or the EF1-tTA in a WAP-Cre-dependent manner (N= 3 biological replicates). Uterine tissues served as positive controls for ERα and PR. GAPDH was used as a loading control.

b. Analysis of KRAS and activation of ERK1/2 on mammary tumors that overexpress ERBB2 (MMTV-neu, N = 3 biological replicates) or oncogenic KRAS in an MMTV-tTA-dependent (N = 3 biological replicates) or EF1-tTA-dependent manner (N = 3 biological replicates).

c. Protein expression of p53, MDM2, and $p19^{Arf}/p16^{Ink4a}$ in claudin-low mammary tumors (N = 3 biological replicates) that originated in WAP-Cre EF1-LSL-tTA TetO-$Kras^{G12D}$ transgenic females in comparison to control cells that lack expression of *Cdkn2a* (NIH3T3) or p53 (p53-/- mouse embryonic fibroblasts, MEFs) or mammary epithelial cells that are known to have a mutation in p53 (HC11).

d. Immunoblot analysis of p53 and $p19^{Arf}/p16^{Ink4a}$ on the tumor specimens shown in panel B.

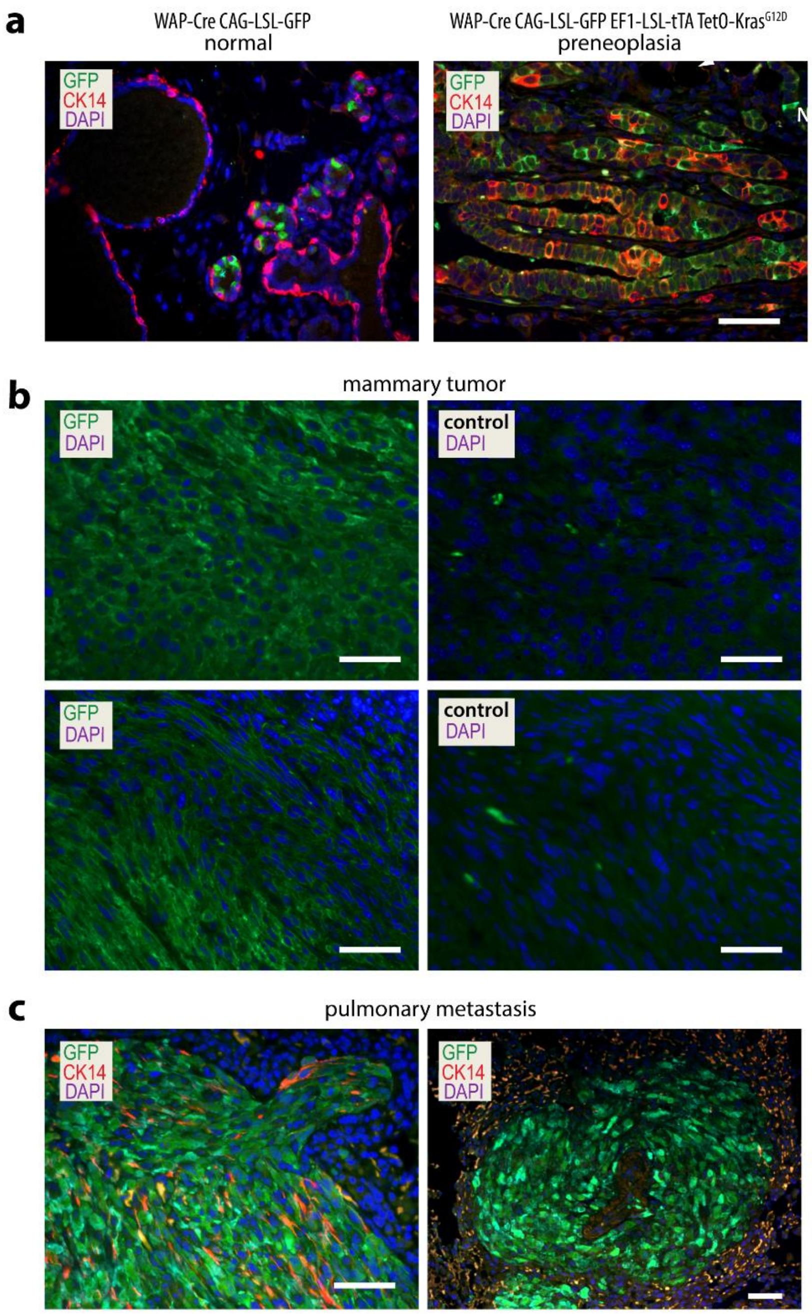

Supplementary Figure 3. Cre/lox-based cell lineage tracing with the CAG-LSL-GFP transgene shows that preneoplastic lesions, mammary tumors with epithelial and mesenchymal appearance, as well as pulmonary metastatic lesions that arise in response to the Cre-mediated and EF1-tTA-driven expression of oncogenic KRAS are derived from luminal cells within terminal ducts that had expressed the WAP-Cre transgene

a. Immunofluorescent staining of GFP in combination with Cytokeratin 14 (CK14) on mammary gland sections from a nonpregnant, parous WAP-Cre CAG-LSL-GFP transgenic control female (left, N = 3 biological replicates) and a small hyperplastic lesion of a parous WAP-Cre CAG-LSL-GFP EF1-LSL-tTA TetO-Kras^{G12D} quadruple transgenic female (right); bars, 50 µm.

b. Immunofluorescent staining of GFP on sections of mammary tumors from WAP-Cre CAG-LSL-GFP EF1-LSL-tTA TetO-Kras^{G12D} transgenic females (N = 6 biological replicates).

c. Immunofluorescent staining of GFP and CK14 on pulmonary metastatic lesions of claudin-low mammary tumors from WAP-Cre CAG-LSL-GFP EF1-LSL-tTA TetO-Kras^{G12D} transgenic females (N = 4 biological replicates).

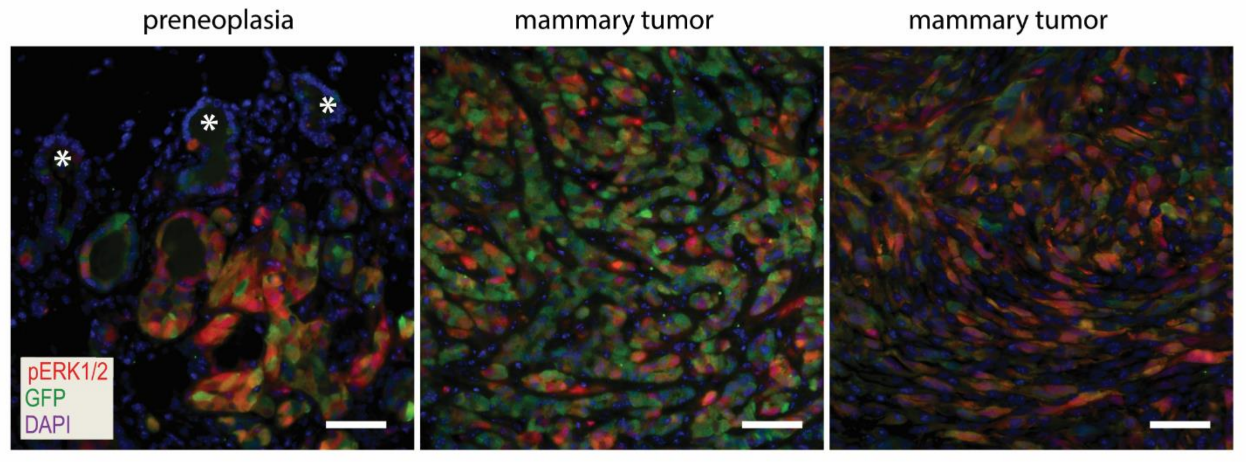

Supplementary Figure 4. The initiation and progression of mutant KRAS-mediated neoplastic transformation and cancer progression is associated with increased MAP kinase phosphorylation

Immunofluorescent staining of pERK1/2 and GFP in preneoplastic lesions (N = 3 biological replicates) as well as early and late-stage mammary cancers of non-pregnant, parous WAP-Cre CAG-LSL-GFP EF1-LSL-tTA TetO-Kras^{G12D} quadruple transgenic females (N = 4 biological replicates); bars, 50 µm. Asterisks indicate the presence of GFP-negative, untransformed mammary epithelial cells within terminal duct lobular units (TDLU) adjacent to GFP-positive, transforming epithelial cells expressing oncogenic KRAS.

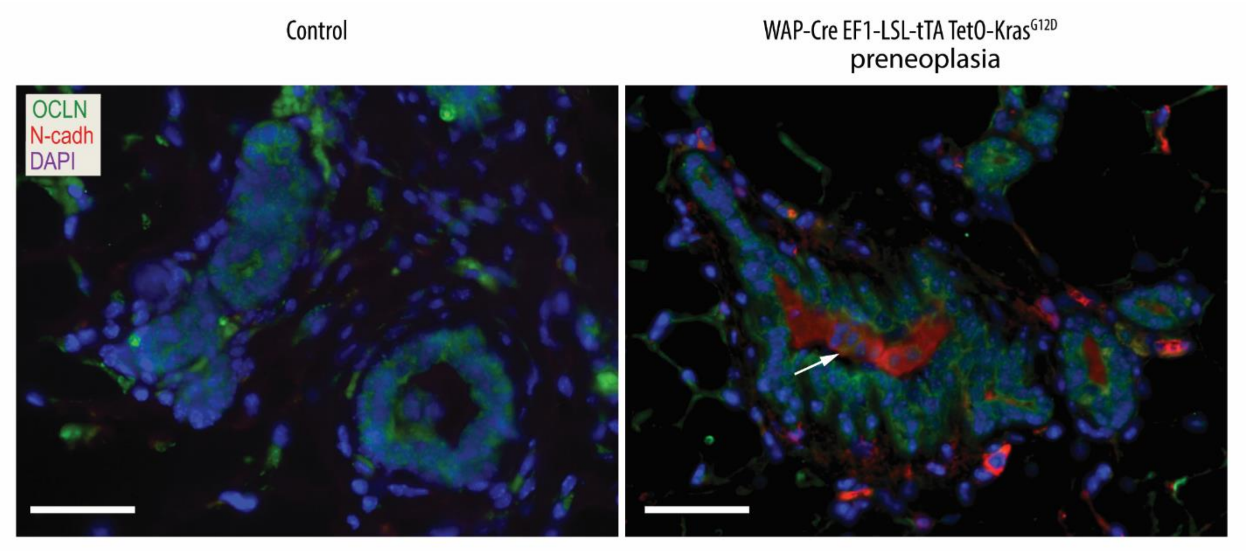

Supplementary Figure 5. Progressive loss of the tight-junction protein Occludin (OCLN) in transforming mammary epithelial cells that acquire N-cadherin expression within early neoplasia of mice that express mutant KRAS in the mammary epithelium

Immunofluorescent staining of Occludin (OCLN) and N-cadherin in the mammary gland sections of WAP-Cre EF1-LSL-tTA TetO-Kras^{G12D} transgenic (N = 3 biological replicates) and wildtype control females; bars, 50 µm. Note that N-cadherin is not present in untransformed mammary epithelial cells that express OCLN (left panel). The arrow in the right panel points to an N-cadherin-positive, transforming epithelial cell that has retained expression of OCLN, which is largely absent in other N-Cadherin-positive cells.

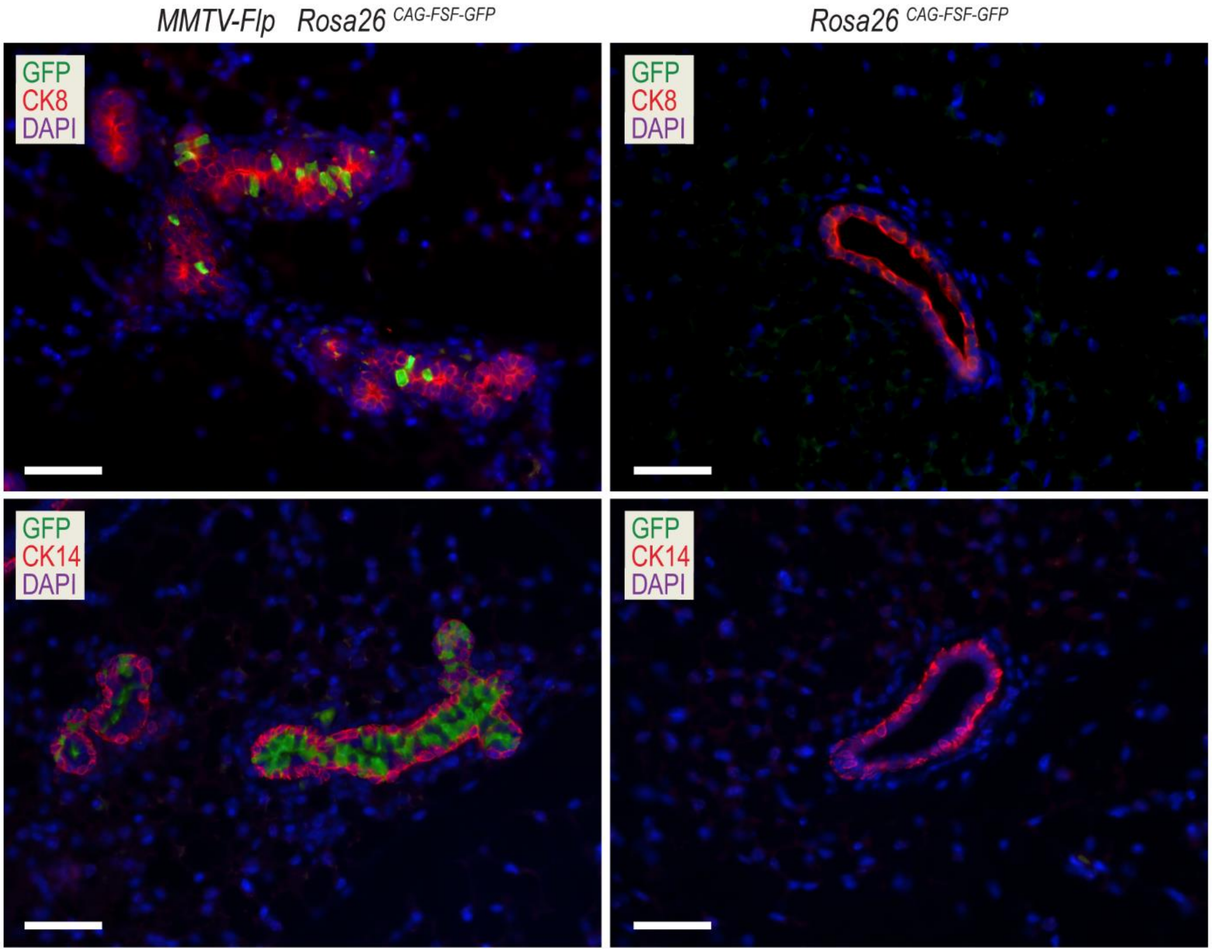

Supplementary Figure 6. The MMTV-Flp transgene is expressed in the luminal epithelium of the mammary gland

Immunofluorescent staining of luminal Cytokeratin 8 (CK8) or basal Cytokeratin 14 (CK14) (red) and GFP (green) on mammary gland sections of MMTV-Flp Rosa26$^{CAG\text{-}FSF\text{-}GFP}$ female mice (N = 2 biological replicates); bars, 50 µm.

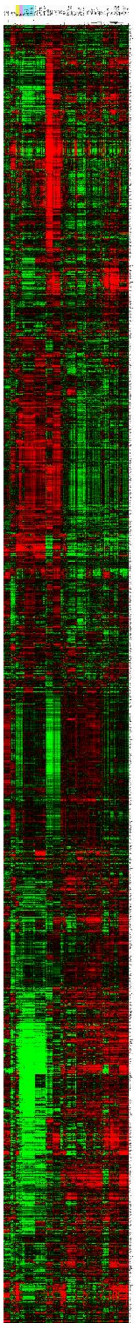

Supplementary Figure 7. The MMTV-Flp-mediated activation of an endogenous *Kras*^{G12D} allele in the mammary epithelium causes basal-like and claudin-low mammary cancers
Complete illustration of the results from the RNA sequencing-based gene expression and cluster analysis that compares the molecular profiles of mammary tumors that originated in MMTV-Flp FSF-*Kras*^{G12D} *p53*^{R172H} females (N= 10 biological replicates) with reference sets from diverse mammary cancer models.

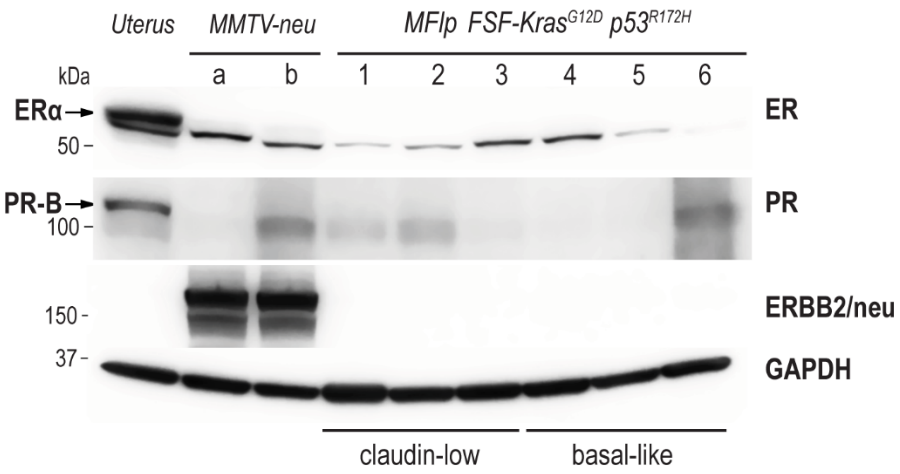

Supplementary Figure 8. Claudin-low and basal-like mammary cancers that originated in females expressing KRAS^{G12D} under the endogenous locus in the mammary epithelium are triple-negative.

Immunoblot analysis of ERα, PR, and ERBB2 on mammary tumors that overexpress wildtype ERBB2/neu (MMTV-neu, samples a and b, N =2 biological replicates) or mutant KRAS from its endogenous *Kras* locus (samples 1-6 correspond to the claudin-low and basal-like tumors shown in the main figure 5, panel E, N = 6 biological replicates). Uterine tissues served as positive controls for ERα and PR. GAPDH was used as a loading control.

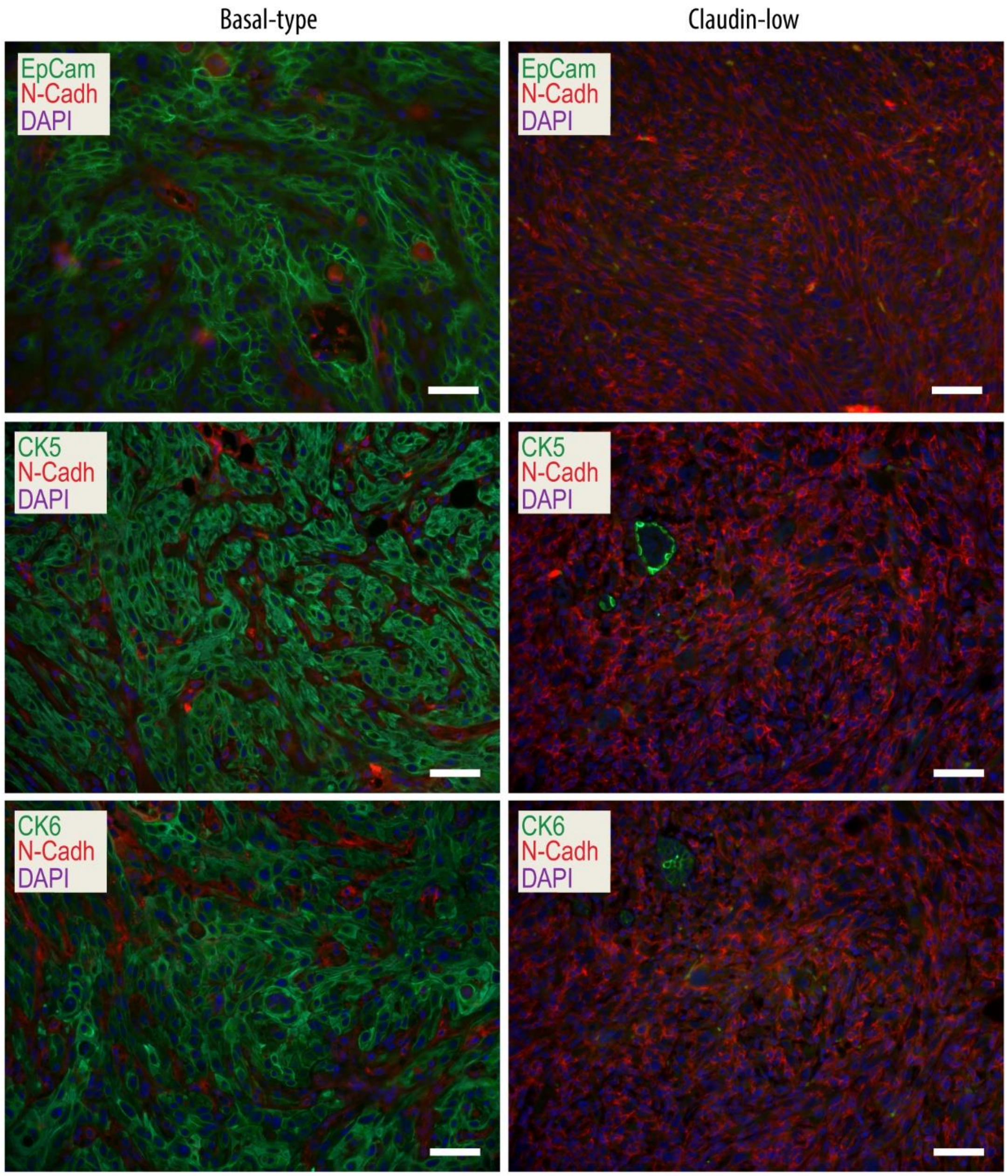

Supplementary Figure 9. Basal-like and claudin-low mammary cancers that arise in female mice expressing oncogenic KRAS^{G12D} from the endogenous *Kras* locus exhibit characteristic changes in the expression of basal keratins, N-Cadherin, as well as EpCAM.
Immunofluorescent staining of EpCAM and basal-type Cytokeratins 5 and 6 (CK5, CK6) in combination with N-Cadherin on sections of basal-like (N = 4 biological replicates) and claudin-low (N = 4 biological replicates) mammary tumors according to their genome-wide gene expression profiles; bars, 50 µm.

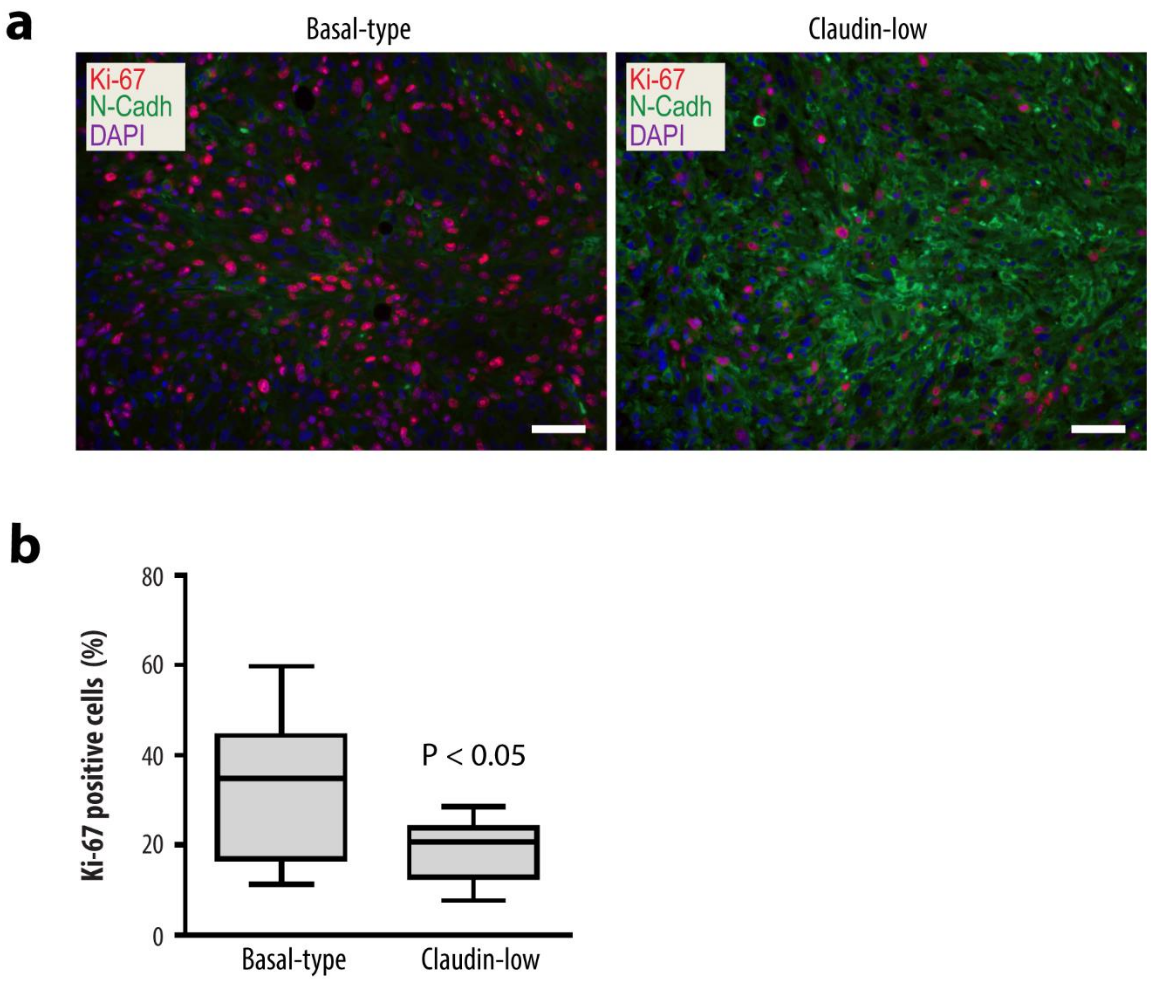

Supplementary Figure 10. Differences in cell proliferation among basal-like and claudin-low mammary cancers expressing oncogenic KRAS^{G12D}

a. Immunofluorescent labeling of Ki-67 and N-Cadherin on sections of basal-like and claudin-low mammary tumors that originated in mice expressing oncogenic KRAS under the control of the endogenous *Kras* locus (N = 4 biological replicates for each subtype).

b. Graphic illustration of the difference in the relative numbers of Ki-67-positive cancer cells basal-like and claudin-low mammary tumors. Ki-67-positive cells in basal-like mammary tumors are graphically represented by minimum = 11.26%, maximum = 59.84%, median = 34.93%, 25th percentile = 16.91%, 75th percentile = 44.34%, and the Ki-67-positive cells in claudin-low mammary tumors were graphically represented by minimum = 7.61%, maximum = 28.52%, median = 20.67%, 25th percentile = 12.81%, 75th percentile = 23.91%. The statistical significance was calculated with a two-sided unpaired t-test, resulting in a *P*-value of <0.05 (*P*-value = 0.0353, t = 2.313, F = 5.850).

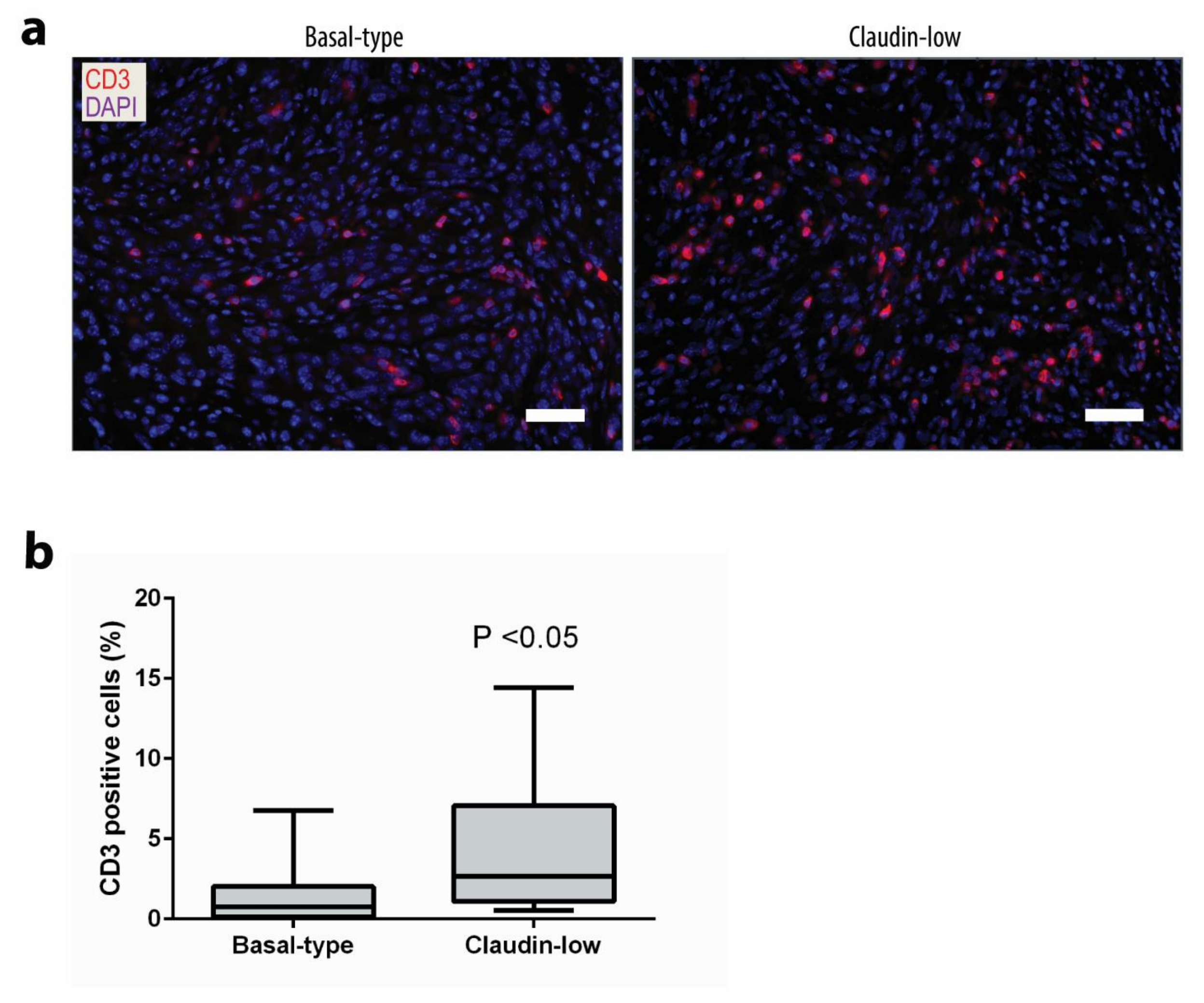

Supplementary Figure 11. More extensive T cell infiltration in claudin-low mammary cancers in comparison to basal-like tumors that originated in females expressing oncogenic $KRAS^{G12D}$

a. Immunofluorescent staining of CD3 within sections of basal-like and claudin-low mammary tumors that originated in mice expressing oncogenic KRAS under the control of the endogenous *Kras* locus (N = 5 biological replicates for each subtype).

b. Graphic illustration of the relative number of T cells among all nucleated cells in basal-like and claudin-low mammary tumors. CD3 positive cells of basal-type mammary tumors are graphically represented by minimum = 0%, maximum = 6.77%, median = 0.77%, 25th percentile = 0.14%, 75th percentile = 2.02%, and CD3 positive cells in claudin-low mammary tumors are graphically represented by minimum = 0.53%, maximum = 14.44%, median = 2.67%, 25th percentile = 1.09%, 75th percentile = 7.10%. The statistical significance was calculated with a two-sided unpaired t-test, resulting in a *P*-value of <0.05 (*P*-value = 0.0427, t = 2.127, F = 3.535).

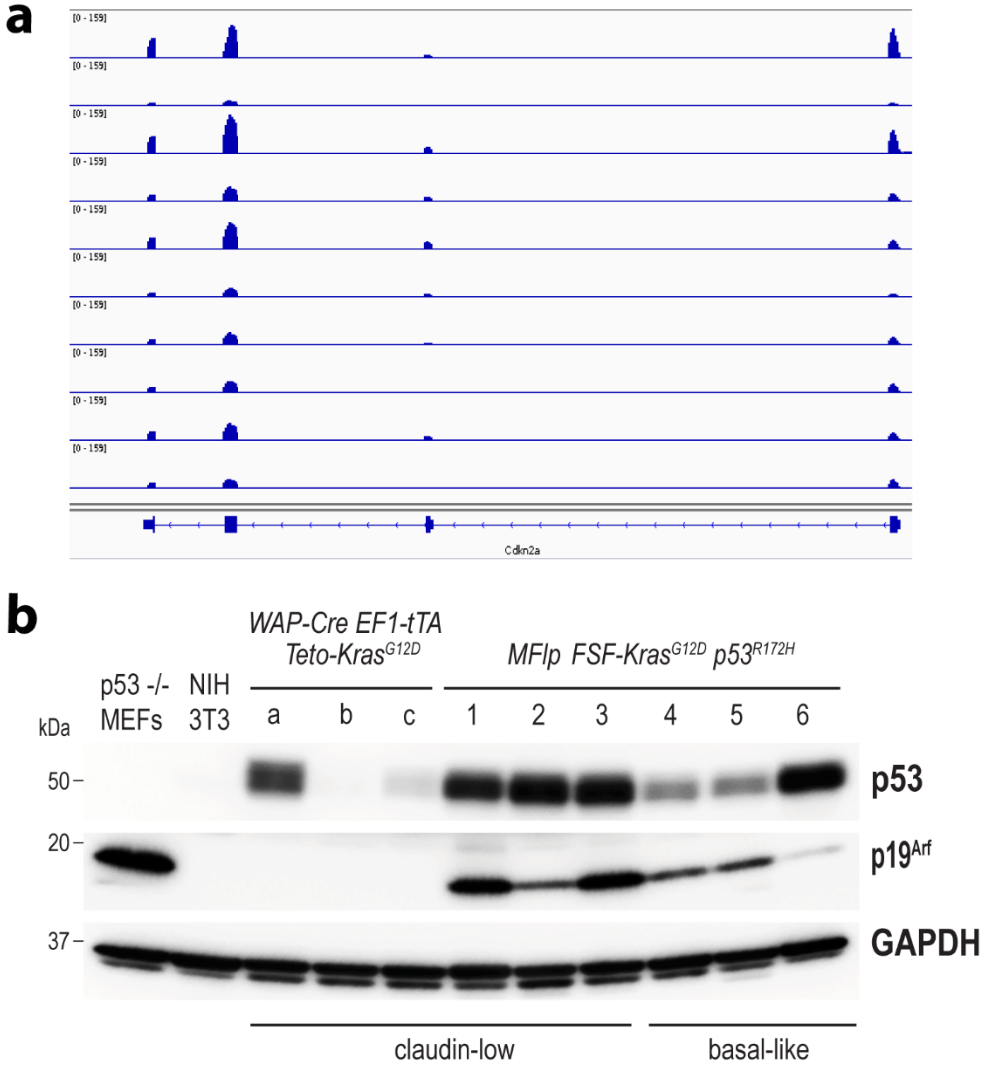

Supplementary Figure 12. Comparative analysis of Cdkn2 and p53 expression in claudin-low mammary tumors that express exogenous or endogenous oncogenic KRAS

a. RNA-sequencing histograms of the *Cdkn2a* locus in mammary cancer tissues from MMTV-Flp FSF-*Kras*^{G12D} *p53*^{R172H} females.

b. Expression p53 and p19^{Arf} proteins in mammary tumors that originated in mice with exogenous mutant KRAS expression (samples a-c) and mice expressing endogenous KRAS^{G12D} (samples 1-6). Samples a-c (N= 3 biological replicates) and 1-3 (N = 3 biological replicates) exhibited claudin-low gene expression signatures and samples 4-6 (N = 3 biological replicates) are basal-like mammary tumors. The matching expression profiles of KRAS, MAPKs, cytokeratins, and Cadherins of these same tumor samples are shown in the main Fig. 5C and 5E.

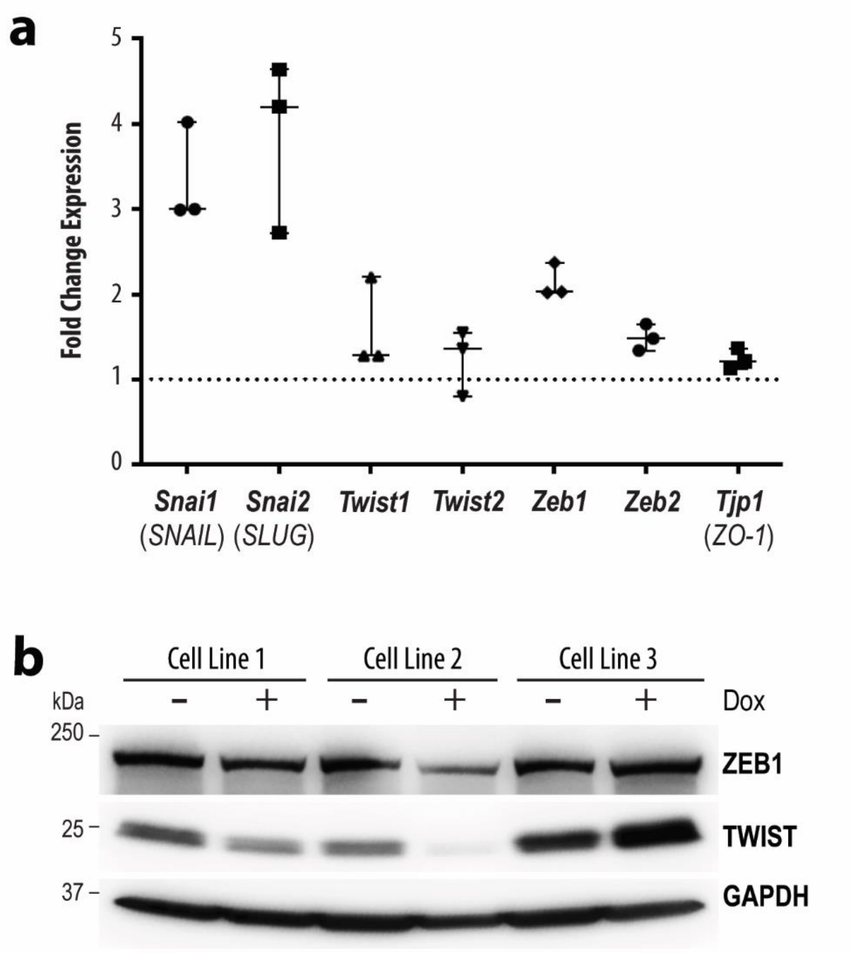

Supplementary Figure 13. Changes in the expression of transcription factors controlling EMT in response to the downregulation of oncogenic KRAS^{G12D} in claudin-low mammary cancer cells

a. Graphic illustration of the changes in mRNA expression of the genes encoding SNAIL (*Snai1*), SLUG (*Snai2*), TWIST1/2, and ZEB1/2 as well as the tight-junction protein ZO-1 (*Tjp1*) following the Dox-mediated suppression of oncogenic KRAS in three independent cell lines (N = 3 biological replicates).

b. Immunoblot analysis to assess the protein expression ZEB1 and TWIST following the Dox-mediated suppression of oncogenic KRAS in the three independent cell lines (N = 3 biological replicates). GAPDH was used as a loading control. It is interesting to note that the protein expression of ZEB1 and TWIST does not follow the general trend of a moderately elevated expression of their genes on the mRNA level following the suppression of oncogenic KRAS as shown in panel a.

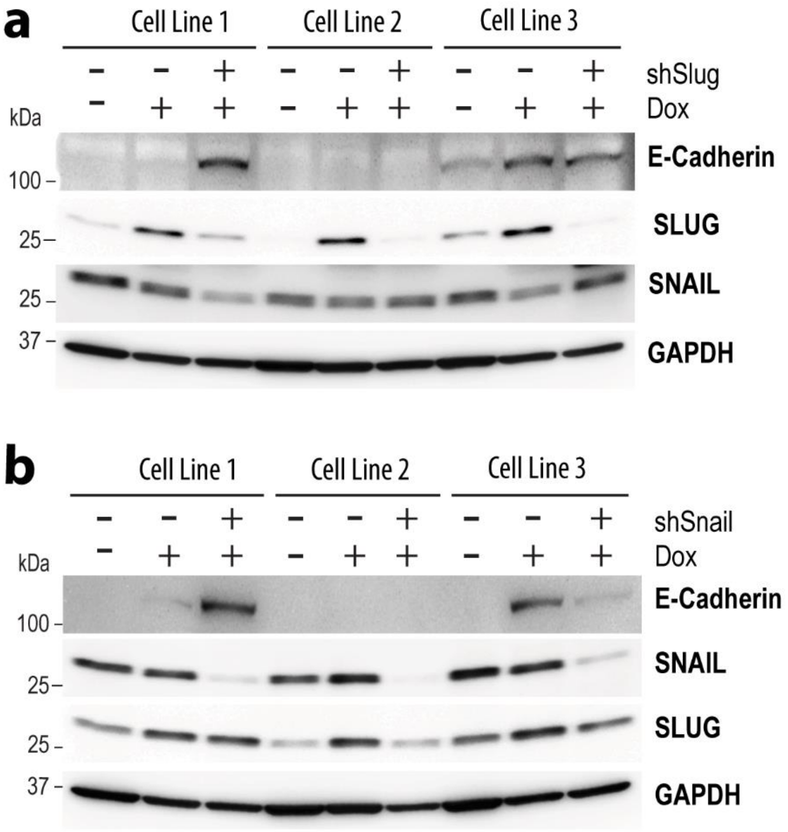

Supplementary Figure 14. Co-contribution of SLUG, SNAIL, and oncogenic RAS in the suppression of E-cadherin in claudin-low mammary cancer cells

a. Immunoblot analysis to assess changes in E-cadherin expression following shRNA-mediated knockdown of SLUG and Dox-mediated suppression of oncogenic KRAS in three independent mammary tumor cell lines (N = 3 biological replicates) from WAP-Cre CAG-LSL-GFP EF1-LSL-tTA TetO-Kras^{G12D} quadruple transgenic females. This is the corresponding blot of the results shown in Fig. 7E using a different short hairpin RNA against SLUG.

b. E-cadherin and SLUG protein expression following shRNA-mediated knockdown of SNAIL (TRCN0000218784) and Dox-mediated suppression of oncogenic KRAS (N = 3 biological replicates).

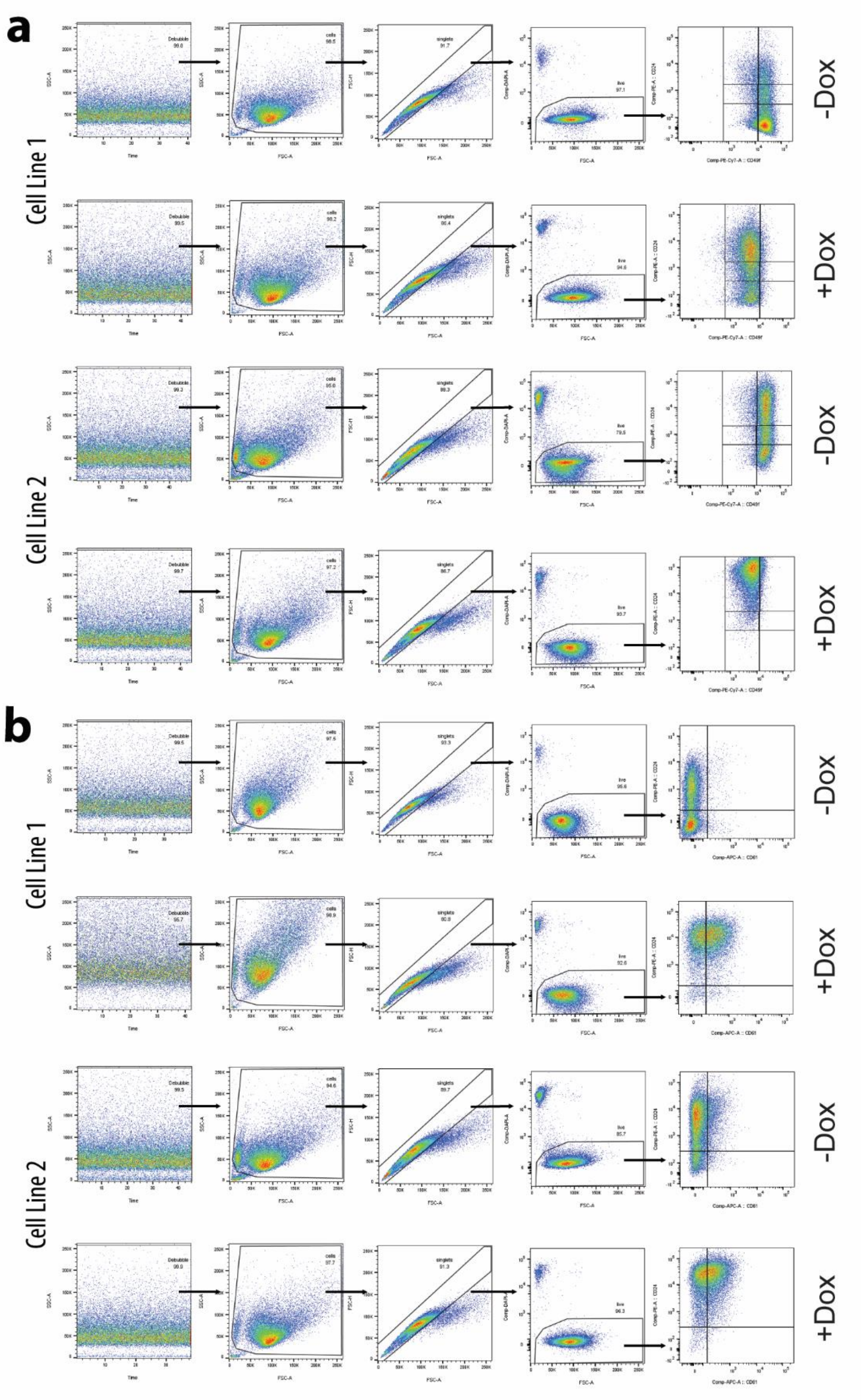

Supplementary Figure 15. Gating strategy for the flow cytometric analysis of CD24, CD49f, and CD61 in claudin-low mammary tumor cells before and after doxycycline-mediated downregulation of oncogenic KRAS

a. Gating strategy for the flow cytometric analysis of CD24-PE and CD49f-PE/Cy7 on untreated and doxycycline (Dox)-treated cell lines shown in Figure 7c, upper panels.

b. Gating strategy for the flow cytometric analysis of CD24-PE and CD61-APC on untreated and doxycycline (Dox)-treated cell lines shown in Figure 7c, lower panels.

Supplementary Materials and Methods

Genotyping

For PCR-based genotyping of mice, genomic DNA was extracted from digested tail biopsies of mice (10 mg/ml proteinase K in 1% SDS, 50nM Tris/HCl pH8, 100nM NaCl, and 50 mM EDTA) using an AutoGenprep2000. The following PCR primer sets were used to genotype the genetically engineered alleles and transgenes.

Supplementary Table 1: PCR primers to genotype transgenes and genetically engineered alleles

Transgene / Allele	Primer #	Sequence (5' - 3')	bp
EF1-LSL-tTA	2453	CGA CTG TGC CTT CTA GTT GCC	315
	2454	AAG AAT GAC TTC CAG CGC CAG GC	
MMTV-tTA	2127	AGT GAT AGA GCT CTT GCC TAG C	364
	599	GCC AAT ACA GTG TAG GCT GC	
WAP-Cre	132	TAG AGC TGT GCC AGC CTC TTC	210
	580	CAT CAC TCG TTG CAT CGA CC	
MMTV-Flp	2546	CCT GGA ACG GCA TCA TCA GC	275
	2128	CTC CCA TTC ATC AGT TCC ATA GG	
CAG-LSL-GFP	2004	GGC TCT AGA GCC TCT GCT AAC C	~270
	2211	GCC ATT GGG ATA TAT CAA CGG TG	
TetO-Kras^{G12D}	2366	GCC TGC GAC GGC GGC ATC TGC	320
	2367	GGG AAT AAG TGT GAT TTG CCT	
TetO-H2B-GFP	811	CCG TCC AGC TCG ACC AGG ATG G	447
	2031	TAC AAC AAG CGC TCG ACC ATC AC	
Rosa26$^{CAG\text{-}FSF\text{-}GFP}$	2729	CCC AAA GTC GCT CTG AGT TGT TAT C	550 (wt)
	2730	GAA GGA GCG GGA GAA ATG GAT ATG	350 (mu)
	2731	CCA GGC GGG CCA TTT ACC GTA AG	
FSF-Kras^{G12D}	2528	CAC CAG CTT CGG CTT CCT ATT	270 (wt)
	2529	AGC TAA TGG CTC TCA AAG GAA TGT A	350 (mu)
	2530	GCG AAG AGT TTG TCC TCA ACC	
Trp53^{R172H}	2598	AGC CTG CCT AGC TTC CTC AGG	290 (wt)
	2599	CTT GGA GAC ATA GCC ACA CTG	330 (mu)

Orthotopic cancer cell transplantation, doxycycline administration, and experimental endpoints

Bilateral orthotopic transplants of mammary tumor fragments or tumor cells into the #4 inguinal mammary glands of 8 to 12 weeks-old female wildtype NCr$^{nu/nu}$ recipients were carried out as described [1]. The experimental endpoint to assess mammary cancer growth in individual genetically engineered mice or transplant recipients was determined by the maximal size of the tumor regardless of the health or condition of an animal. The maximal allowed tumor size was approximately 1.5 cm in diameter as mandated by the Institutional Animal Care and Use Committees. To suppress the expression of exogenous mutant KRAS in established mammary tumors *in vivo*, recipient mice that carry cancer tissues from MMTV-tTA TetO-Kras^{G12D} or WAP-Cre EF1-LSL-tTA TetO-Kras^{G12D} females were fed doxycycline (Dox, Sigma D9891-25G) in their drinking water at a concentration of 2 mg/ml supplemented with.50 mg/ml sucrose for durations shown in the *Results* section. Tumors were measured using an electronic caliper, and tumor volumes (mm^3) were calculated using the following equation: length (mm) x width^2 (mm^2)/2.

Immunostaining

Tissues were fixed overnight at room temperature in 10% buffered formalin (Fisher Scientific Company) and embedded in paraffin by standard methods. Histologic sections of 5µm were deparaffinized three times in 100% Histo-Clear, rehydrated in decreasing concentrations of ethanol (100%, 95%, 90%, 70%, 50%, and 30% for 3 minutes each), and washed for 5 min in 1x PBS. For antigen retrieval and immunostaining, tissue sections were pressure cooked in ImmunoRetriever with Citrate (Bio SB) using a Bio SB TintoRetriever pressure cooker, which was set to 116-121°C for 4 minutes. Once the slides had returned to room temperature, they were rinsed for 5 min in 1x PBS and blocked with 3% BSA for 1h. Subsequently, primary antibodies were added (varying dilutions, staining conditions can be provided upon request) and incubated overnight at 4°C in a moist chamber. The following day the slides were washed three times with 1x PBS for 5 minutes each, a fluorophore-conjugated secondary antibody was added, and slides were incubated in the dark for 1h at room temperature in a moist chamber. After washing the slides twice with distilled water, Vectashield DAPI mounting media (Vector, H-1200) and coverslips were applied.

Supplementary Table 2: Primary and secondary antibodies for immunostaining

**Primary Antibodies**	**SOURCE**	**IDENTIFIER**	**DILUTION**
α-GFP	Avēs Labs	GFP-1020	1:1000
α-CK8	Developmental Studies Hybridoma Bank	TROMAI	1:100
α-CK14	Covance	PRB-155P	1:1000
α-pERK1/2	Cell Signaling	#9101S	1:250
α-EpCAM	Cell Signaling	#93790S	1:200
α-E-cadherin	Cell Signaling	#3195	1:200
α-N-cadherin	Cell Signaling	#14215	1:200
α-Occludin	Invitrogen	71-1500	1:300
α-CD3	Cell Signaling	#78588S	1:200
α-CK5	Covance	PRB-160P	1:200
α-CK6	Covance	PRB-169P	1:200
α-Ki67	Abcam	ab15580	1:300
**Secondary Antibodies**	**SOURCE**	**IDENTIFIER**	**Dilution**
Alexa Fluor 488 goat anti-chicken	Invitrogen	A11039	1:1000
Alexa Fluor 488 donkey anti-rat	Invitrogen	A21208	1:1000
Alexa Fluor 594 goat anti-rat	Invitrogen	A11007	1:1000
Alexa Fluor 594 goat anti-rabbit	Invitrogen	A11012	1:1000
Alexa Fluor 594 donkey anti-rabbit	Invitrogen	A21207	1:1000
Alexa Fluor 488 donkey anti-rabbit	Invitrogen	A21206	1:1000
Alexa Fluor 594 goat anti-mouse	Invitrogen	A11005	1:1000
Alexa Fluor 488 donkey anti-mouse	Invitrogen	A21202	1:1000

Knockdown of the SNAIL and SLUG proteins in mouse mammary tumor cells

To knock down the SNAIL protein, we constructed two lentiviral gene transfer vectors by cloning small hairpin sequences (TRCN0000218784 and TRCN0000234034) into the *Age*1 and *Eco*R1 sites of the pLKO.1neo vector. The pLKO.1neo was a gift from Sheila Stewart to Addgene (#13425).

TRCN0000218784 (5'-CCG GAT GTG TCT CCC AGA ACT ATT TCT CGA GAA ATA GTT CTG GGA GAC ACA TTT TTT G-3' and 5'- AAT TCA AAA AAT GTG TCT CCC AGA ACT ATT TCT CGA GAA ATA GTT CTG GGA GAC ACA T-3')

TRCN0000234034 (5'-CCG GGA TCT TCA ACT GCA AAT ATT GCT CGA GCA ATA TTT GCA GTT GAA GAT CTT TTT G-3' and 5'- AAT TCA AAA AGA TCT TCA ACT GCA AAT ATT GCT CGA GCA ATA TTT GCA GTT GAA GAT C-3')

The correct sequences of the *Snail* (*Snai1*) shRNAs were validated by Sanger sequencing prior to the production of lentiviral particles. Mammary cancer cells were infected with lentiviral particles and selected with 400 µg/ml Geneticin® (Gibco 10131-035).

To knock down SLUG protein, we obtained two previously validated pLKO.1puro shSlug (*shSnai2*) constructs from Addgene (#40647, #40648) [2]. Following infection with lentiviral particles, cell we selected with 7 µg/ml puromycin.

Immunoblot analyses

Cell pellets or homogenized tissues sonicated for 3 seconds in complete lysis buffer containing 1% Nonidet P-40, 0.5% sodium deoxycholate, 0.1% SDS, 1 mM phenylmethylsulfonyl fluoride, 0.4 units/ml aprotinin, 1 mM NaF, leupeptin, and 0.1 mM sodium orthovanadate and kept on ice for 30 minutes. Whole-cell extracts were resolved by SDS-PAGE and blotted onto polyvinylidene fluoride (PVDF) membranes (Invitrogen). The membranes were blocked for 1 h at room temperature in 5% dry milk in 1x TBST (Tris-buffered saline with 0.05% Tween-20) buffer or in 5% Bovine Serum Albumin (BSA) in 1x TBST for phosphotyrosine-specific antibodies. Subsequently, membranes were incubated with primary antibodies in blocking buffer at 4 ºC overnight. Next day, membranes were washed three times for 5 min in 1x TBST and incubated for 1h at room temperature with horseradish peroxidase-conjugated secondary antibodies [goat anti-rabbit (HAF008) from R&D Systems or Digital anti-Mouse-HRP (R1005) from KwikQuant] in blocking buffer. Membranes were washed five times for 5 min with 1x TBST and then two times for 5 min in 1x TBS (Tris-buffered saline without Tween 20) and finally for 5 min in ultrapure water (Invitrogen 10977-023). Protein bands were detected using the ECL chemiluminescence kit for Western blot analysis [KwikQuant Ultra Digital-ECLTM Substrate Solution (Cat #: R1002)] according to the instructions by the manufacturer (Kindle Biosciences, LCC). Membranes were stripped using a mild glycine stripping buffer (Abcam protocol) for consecutive detection of various proteins.

Supplementary Table 3: Primary and secondary antibodies for immunoblotting

**Primary Antibodies**	**SOURCE**	**IDENTIFIER**	**DILUTION**
α-KRAS	Abcam	ab180772	1:2000
α-pERK1/2	Cell Signaling	#9101S	1:1000
α-ERK1/2	BD Transduction Laboratories	#610123	1:1000
α-GAPDH	Cell Signaling	#5174S	1:10000
α-E-cadherin	Cell Signaling	#3195	1:1000
α-N-cadherin	Cell Signaling	#14215	1:1000
α-EpCAM	Cell Signaling	#93790S	1:1000
α-CK14	Covance	PRB-155P	1:1000
α-ER	Abcam	ab32063	1:1000
α-PR	Abcam	ab2765	1:1000
α-cleaved Caspase 3	Cell Signaling	#9661S	1:1000
α-ITGB3 (CD61)	Abcam	ab75872	1:1000
α-GATA3	Cell Signaling	#5852T	1:1000
α-SLUG	Cell Signaling	#9585T	1:1000
α-SNAIL	Cell Signaling	#3879T	1:1000
α-TWIST	Santa Cruz	sc81417	1:1000
α-ZEB1	Cell Signaling	#70512T	1:1000
α-ERBB2	Calbiochem	OP-15	1:1000
α-p53	Cell Signaling	#2524T	1:1000
α-MDM2	Oncogene	OP115T-10UG	1:1000
α-p19^{Arf}/p16^{Ink4a}	Abcam	ab80	1:1000
**Secondary Antibodies**	**SOURCE**	**IDENTIFIER**	**DILUTION**
HRP-conjugated goat anti-rabbit	R&D Systems	HAF008	1:1000
Digital anti-Mouse-HRP	KwikQuant	R1005	1:1000

Fold change determination for EMT genes

To determine the fold changes of the EMT genes the CPM value of the Dox-treated cell line was divided by the CPM value of its isogenic untreated cell line for the tree isogenic cell lines. The resulting values were plotted with GraphPad Prism 6 software (v6.07; GraphPad Software, Inc., La Jolla, CA). The plots are represented as box and whiskers with indication of the minimum and maximum value, as well as first quartile, median, and third quartile. All data points are individually displayed.

References

1 Sakamoto, K., Lin, W. C., Triplett, A. A. & Wagner, K. U. Targeting janus kinase 2 in Her2/neu-expressing mammary cancer: Implications for cancer prevention and therapy. *Cancer Res.* **69**, 6642-6650 (2009).

2 Guo, W. *et al.* Slug and Sox9 cooperatively determine the mammary stem cell state. *Cell* **148**, 1015-1028, doi:10.1016/j.cell.2012.02.008 (2012).
