# Supplementary material for: Highly metastatic claudin-low mammary cancers can originate from luminal epithelial cells
Source: Nat Commun. 2021 Jun 18;12:3742. doi: 10.1038/s41467-021-23957-5 (PMC8213728; doi:10.1038/s41467-021-23957-5)
Supplement: Supplementary file 3 — Description of Additional Supplementary Files [file 41467_2021_23957_MOESM3_ESM.docx]

**File Name:** Supplementary Data 1

**Description:** RNA-Sequencing data sets from reference mammary tumor models
