# Supplementary material for: Highly metastatic claudin-low mammary cancers can originate from luminal epithelial cells
Source: Nat Commun. 2021 Jun 18;12:3742. doi: 10.1038/s41467-021-23957-5 (PMC8213728; doi:10.1038/s41467-021-23957-5)
Supplement: Supplementary file 5 — Reporting Summary [file 41467_2021_23957_MOESM5_ESM.pdf]

nature research

Corresponding author(s): Kay-Uwe Wagner

Last updated by author(s): May 4, 2021

Reporting Summary

Nature Research wishes to improve the reproducibility of the work that we publish. This form provides structure for consistency and transparency in reporting. For further information on Nature Research policies, see our Editorial Policies and the Editorial Policy Checklist.

Statistics

For all statistical analyses, confirm that the following items are present in the figure legend, table legend, main text, or Methods section.

n/a	Confirmed	
☐	☒	The exact sample size (*n*) for each experimental group/condition, given as a discrete number and unit of measurement
☐	☒	A statement on whether measurements were taken from distinct samples or whether the same sample was measured repeatedly
☐	☒	The statistical test(s) used AND whether they are one- or two-sided<br>*Only common tests should be described solely by name; describe more complex techniques in the Methods section.*
☒	☐	A description of all covariates tested
☐	☒	A description of any assumptions or corrections, such as tests of normality and adjustment for multiple comparisons
☐	☒	A full description of the statistical parameters including central tendency (e.g. means) or other basic estimates (e.g. regression coefficient) AND variation (e.g. standard deviation) or associated estimates of uncertainty (e.g. confidence intervals)
☐	☒	For null hypothesis testing, the test statistic (e.g. *F*, *t*, *r*) with confidence intervals, effect sizes, degrees of freedom and *P* value noted<br>*Give P values as exact values whenever suitable.*
☒	☐	For Bayesian analysis, information on the choice of priors and Markov chain Monte Carlo settings
☐	☒	For hierarchical and complex designs, identification of the appropriate level for tests and full reporting of outcomes
☒	☐	Estimates of effect sizes (e.g. Cohen's *d*, Pearson's *r*), indicating how they were calculated

Our web collection on statistics for biologists contains articles on many of the points above.

Software and code

Policy information about availability of computer code

Data collection	The flow cytometric data was acquired on a BD Biosciences LSRII (FACSdiva 8.0.1). The RNA expression library construction and next generation sequencing was performed by Novogene Corp. using a Novaseq 6000.
Data analysis	Following software was used in this study as described in the manuscript: FACSdiva (v8.0.1); Fiji (v 2.0.0); GraphPad Prism6 (v6.07); FastQC (v0.11.9); R (version 3.6.0); Rsubread (v2.0.0); edgeR (v3.28.0); clusterProfiler (v3.0.4); gplots (v3.0.3); IGV (v2.7.2); Cluster3.0 (v3.0).<br>For RNA Sequencing, the quality of sequenced reads was determined using FastQC (http://www.bioinformatics.babraham.ac.uk/projects/fastqc, v0.11.9). For differential expression analyses the 150 base pair paired-end reads were mapped to the mm10 mouse reference genome with Rsubread (v2.0.0). Transcript abundance was determined using featureCounts from the Rsubread package. Low abundance transcripts (cpm <=5) that occurred in more than half of the samples were excluded from subsequent analyses. The R package edgeR (v3.28.0) was used to normalize the transcript counts and to perform differential expression analysis of paired samples (i.e., expression analyses of the same cells on and off doxycycline). Transcript abundance was estimated in counts per million (cpm). Before the differential expression analysis was performed genes that showed expression below 5 cpm in more than half of the samples were excluded from the analysis. Genes that showed differential expression by more than 2-fold and an FDR (False Discovery Rate) of below 0.001 between the sample groups were considered as significantly deregulated. The gene set enrichment analysis was performed with R-function gseKEGG and plotted with the function R-gseaplot (package: clusterProfiler v3.0.4). The corresponding heatmaps were created by log2 transforming the count data and by extracting the significantly deregulated genes that belong to the corresponding pathways. Heatmaps were plotted with the R-function heatmap.2 (package: gplots v3.0.3)(http://cran.r-project.org/web/packages/gplots/index.html). The Broad Institute's Integrative Genomics Viewer (IGV v2.7.2) was used to visualize the expression of individual genes and their exons.

For manuscripts utilizing custom algorithms or software that are central to the research but not yet described in published literature, software must be made available to editors and reviewers. We strongly encourage code deposition in a community repository (e.g. GitHub). See the Nature Research guidelines for submitting code & software for further information.

Data

Policy information about availability of data

All manuscripts must include a data availability statement. This statement should provide the following information, where applicable:

- Accession codes, unique identifiers, or web links for publicly available datasets
- A list of figures that have associated raw data
- A description of any restrictions on data availability

The new RNA Sequencing data was deposited in the Gene Expression Omnibus (GEO) under accession number GSE157333. Supplemental Table S1 in the Supplemental Material and Methods provides a detailed list of RNA Sequencing data sets from reference mammary tumor models. The GEO data sets associated with Supplementary Table S1 are GSE118164, GSE124821, and GSE148482. Microarray data sets from reference mammary tumor models are available under GEO accession numbers GSE3165, GSE8516, GSE9343, GSE14457, GSE15263, GSE17916, GSE27101, and GSE42640. The raw data from these sources are associated with Figures 2, 5D, 6E, and Supplemental Figure S7.

The data sets with associated links:
GSE157333 [https://www.ncbi.nlm.nih.gov/geo/query/acc.cgi?acc=GSE157333]
GSE3165 [https://www.ncbi.nlm.nih.gov/geo/query/acc.cgi?acc=GSE3165]
GSE8516 [https://www.ncbi.nlm.nih.gov/geo/query/acc.cgi?acc=GSE8516]
GSE9343 [https://www.ncbi.nlm.nih.gov/geo/query/acc.cgi?acc=GSE9343]
GSE14457 [https://www.ncbi.nlm.nih.gov/geo/query/acc.cgi?acc=GSE14457]
GSE15263 [https://www.ncbi.nlm.nih.gov/geo/query/acc.cgi?acc=GSE15263]
GSE17916 [https://www.ncbi.nlm.nih.gov/geo/query/acc.cgi?acc=GSE17916]
GSE27101 [https://www.ncbi.nlm.nih.gov/geo/query/acc.cgi?acc=GSE27101]
GSE42640 [https://www.ncbi.nlm.nih.gov/geo/query/acc.cgi?acc=GSE42640]
GSE118164 [https://www.ncbi.nlm.nih.gov/geo/query/acc.cgi?acc=GSE118164]
GSE124821 [https://www.ncbi.nlm.nih.gov/geo/query/acc.cgi?acc=GSE124821]
GSE148482 [https://www.ncbi.nlm.nih.gov/geo/query/acc.cgi?acc=GSE148482]

Field-specific reporting

Please select the one below that is the best fit for your research. If you are not sure, read the appropriate sections before making your selection.

☒ Life sciences ☐ Behavioural & social sciences ☐ Ecological, evolutionary & environmental sciences

For a reference copy of the document with all sections, see nature.com/documents/nr-reporting-summary-flat.pdf

Life sciences study design

All studies must disclose on these points even when the disclosure is negative.

Sample size	As a tool for an initial calculation of the number of animals, we used the sample size calculator from Dr. La Morte the Boston University Medical Center. To calculate the minimum number of mice for each experiment outlined in this application, we use a P value of 0.05 and a power level of 80-90%.The mean tumor latencies and their variations (i.e., standard deviations) of the new cancer models were obtained from preliminary data of unpublished experiments performed by our team. Since the variability among selected new intercrosses was unknown (nobody had done these experiments), we assumed that the standard deviation within these groups was at least as large as that in the pilot studies, and a difference in tumor latency among experimental groups and their controls of at least one month was estimated as biologically meaningful.
Data exclusions	No data was excluded
Replication	The conclusions drawn in this manuscript are based on three different mouse models and in vitro cell line studies . For each model, the conclusions are supported by at least 4 biological replicates. The mouse studies are further supported by in vitro cell line work that additionally includes at least 3 biological replicates. Consistent results across biological and technical replicates support the conclusions that were drawn. Replication was successful.
Randomization	Randomization into experimental groups was not relevant for this study. Experimental mice and litter-mate controls were determined based on the desired genotype, i.e. the presence of transgenes and targeted gene loci that facilitated the development of mammary tumors. Litter-mate controls that did not carry all the necessary transgenes for tumor induction were analyzed as controls. This is a mammary tumor study, and consequently only females mice were used. In one line of investigation using WAP-Cre EF1-tTA TetO-Kras mice, experimental and control females with the same genotype were compared as multiparous (experimental) and virgin (nulliparous controls). For the study of molecular and cellular characteristics of claudin-low and basal-like mammary cancer, tumors were selected based on the genome-wide gene expression profiles. For molecular studies on the effects of the downregulation of KRAS in mammary cancer cells, we used pairs of three cell lines, i.e., untreated controls and treated with Dox to downregulate KRAS.
Blinding	Investigators were blinded to group allocation determining the proliferation rates and numbers of CD3 cells in basal-like versus claudin-low mammary cancer. In all other studies, blinding was not relevant as the genotypes of experimental animals and controls, their parity status, as well as the genotype of derived cell lines was known to the investigators. The preceding genotyping was performed using unbiased assays (PCR) and always included positive and negative controls for all transgenes and for all animals used in this study. Data acquisition on animals

and cell lines was performed on scientific equipment (i.e., sequencing machines, microarray readers, imaging tools), and the resulting data was analyzed and quantified through computational approaches.

Reporting for specific materials, systems and methods

We require information from authors about some types of materials, experimental systems and methods used in many studies. Here, indicate whether each material, system or method listed is relevant to your study. If you are not sure if a list item applies to your research, read the appropriate section before selecting a response.

Materials & experimental systems

n/a	Involved in the study
☐	☒ Antibodies
☐	☒ Eukaryotic cell lines
☒	☐ Palaeontology and archaeology
☐	☒ Animals and other organisms
☒	☐ Human research participants
☒	☐ Clinical data
☒	☐ Dual use research of concern

Methods

n/a	Involved in the study
☒	☐ ChIP-seq
☐	☒ Flow cytometry
☒	☐ MRI-based neuroimaging

Antibodies

Antibodies used

List of primary and secondary antibodies for immunostaining
Primary Antibodies; SOURCE; IDENTIFIER
α-GFP; Avēs Labs; GFP-1020
α-CK8; Developmental Studies Hybridoma Bank; TROMAI
α-CK14; Covance; PRB-155P
α-E-cadherin; Cell Signaling; #3195
α-N-cadherin; Cell Signaling; #14215
α-CK5; Covance; PRB-160P
α-CK6; Covance; PRB-169P
α-Ki67; Abcam; ab15580
α-pERK1/2; Cell Signaling; #9101S
α-EpCAM; Cell Signaling; #93790S
α-Occludin; Invitrogen; 71-1500
α-CD3; Cell Signaling; #78588S

Secondary Antibodies; SOURCE; IDENTIFIER
Alexa Fluor 488 goat anti-chicken; Invitrogen; A11039
Alexa Fluor 488 donkey anti-rat; Invitrogen; A21208
Alexa Fluor 594 goat anti-rat; Invitrogen; A11007
Alexa Fluor 594 goat anti-rabbit; Invitrogen; A11012
Alexa Fluor 594 donkey anti-rabbit; Invitrogen; A21207
Alexa Fluor 488 donkey anti-rabbit; Invitrogen; A21206
Alexa Fluor 594 goat anti-mouse; Invitrogen; A11005
Alexa Fluor 488 donkey anti-mouse; Invitrogen; A21202

List of primary and secondary antibodies for immunoblotting

Primary Antibodies; SOURCE; IDENTIFIER
α-KRAS; Abcam; ab180772
α-pERK1/2; Cell Signaling; #9101S
α-ERK1/2; BD Transduction Laboratories; #610123
α-GAPDH; Cell Signaling; #5174S
α-E-cadherin; Cell Signaling; #3195
α-N-cadherin; Cell Signaling; #14215
α-EpCAM; Cell Signaling; #93790S
α-CK14; Covance; PRB-155P
α-cleaved Caspase 3; Cell Signaling; #9661S
α-ITGB3 (CD61); Abcam; ab75872
α-GATA3; Cell Signaling; #5852T
α-SLUG; Cell Signaling; #9585T
α-SNAIL; Cell Signaling; #3879T
α-ERBB2; Calbiochem; OP-15
α-p53; Cell Signaling; #2524T

α-MDM2; Oncogene; OP115T-10UG
α-p19Arf/p16Ink4a; Abcam; ab80
α-ER; Abcam; ab32063
α-PR; Abcam; ab2765
α-TWIST; Santa Cruz; sc81417
α-ZEB1; Cell Signaling; #70512T

Secondary Antibodies; SOURCE; IDENTIFIER
HRP-conjugated goat anti-rabbit; R&D Systems; HAF008
Digital anti-Mouse-HRP; KwikQuant; R1005

Flow cytometry Antibodies:
PE Rat Anti-Mouse CD24; BD PharmingenTM; 553262
PE/Cy7 Rat Anti-Human/Mouse CD49f; BioLegend; 313621
APC Armenian hamster Anti-Mouse CD61; Thermo Fisher Scientific; MCD6105

Validation

List of primary and secondary antibodies and their validation for immunostaining on mouse tissues. The list includes information provided by the manufactures as well as relevant publications for each antibody, application, species specificity.

Primary Antibodies; SOURCE; IDENTIFIER

α-GFP; Avēs Labs; GFP-1020: "Antibodies were analyzed by western blot analysis (1:5000 dilution) and immunohistochemistry (1:500 dilution) using transgenic mice expressing the GFP gene product" (https://www.aveslabs.com/products/green-fluorescent-protein-gfp-antibody). Also see: Sakamoto, K., Rädler, P.D., Wehde, B.L. et al. Efficient tissue-type specific expression of target genes in a tetracycline-controlled manner from the ubiquitously active Eef1a1 locus. Sci Rep 10, 207 (2020). https://doi.org/10.1038/s41598-019-57052-z

α-CK8; Developmental Studies Hybridoma Bank; TROMAI. Antibody was validated for IF in several publications (see: https://dshb.biology.uiowa.edu/TROMA-I). Also see: Leung AW, Kent Morest D, Li JY. Differential BMP signaling controls formation and differentiation of multipotent preplacodal ectoderm progenitors from human embryonic stem cells. Dev Biol. 2013;379(2):208-220. doi:10.1016/j.ydbio.2013.04.023.

α-CK14; Covance; PRB-155P: "Each lot of this antibody is quality control tested by immunohistochemical staining" (https://www.biolegend.com/en-us/products/keratin-14-polyclonal-antibody-purified-10953). Also see: Nair SJ, Zhang X, Chiang HC, et al. Genetic suppression reveals DNA repair-independent antagonism between BRCA1 and COBRA1 in mammary gland development. Nat Commun. 2016;7:10913. Published 2016 Mar 4. doi:10.1038/ncomms10913

α-E-cadherin; Cell Signaling; #3195: Certificate of analysis states "This document certifies that this product has met all of the quality control standards defined by Cell Signaling Technology, Inc." (https://www.cellsignal.com/products/primary-antibodies/e-cadherin-24e10-rabbit-mab/3195). Also see: Nikolopoulou E, Hirst CS, Galea G, et al. Spinal neural tube closure depends on regulation of surface ectoderm identity and biomechanics by Grhl2. Nat Commun. 2019;10(1):2487. Published 2019 Jun 6. doi:10.1038/s41467-019-10164-6

α-N-cadherin; Cell Signaling; #14215: Certificate of analysis states "This document certifies that this product has met all of the quality control standards defined by Cell Signaling Technology, Inc." (https://www.cellsignal.com/products/primary-antibodies/n-cadherin-13a9-mouse-mab/14215?site-search-type=Products&N=4294956287&Ntt=%2314215&fromPage=plp&_requestid=512665). Also see: Nikolopoulou E, Hirst CS, Galea G, et al. Spinal neural tube closure depends on regulation of surface ectoderm identity and biomechanics by Grhl2. Nat Commun. 2019;10(1):2487. Published 2019 Jun 6. doi:10.1038/s41467-019-10164-6

α-CK5; Covance; PRB-160P: "Each lot of this antibody is quality control tested by immunohistochemical staining" (https://www.biolegend.com/en-us/products/keratin-5-polyclonal-antibody-purified-10956). Also see: Tadokoro T, Gao X, Hong CC, Hotten D, Hogan BL. BMP signaling and cellular dynamics during regeneration of airway epithelium from basal progenitors. Development. 2016;143(5):764-773. doi:10.1242/dev.126656

α-CK6; Covance; PRB-169P: "Each lot of this antibody is quality control tested by immunohistochemical staining" (https://www.biolegend.com/en-us/products/purified-anti-mouse-keratin-6a-antibody-11459). Also see: DiTommaso T, Cottle DL, Pearson HB, et al. Keratin 76 is required for tight junction function and maintenance of the skin barrier. PLoS Genet. 2014;10(10):e1004706. Published 2014 Oct 23. doi:10.1371/journal.pgen.1004706.

α-Ki67; Abcam; ab15580: "Knockout validated" (https://www.abcam.com/ki67-antibody-ab15580.html). Also see: Parfitt, G.J. Immunofluorescence Tomography: High-resolution 3-D reconstruction by serial-sectioning of methacrylate embedded tissues and alignment of 2-D immunofluorescence images. Sci Rep 9, 1992 (2019). https://doi.org/10.1038/s41598-018-38232-9.

α-pERK1/2; Cell Signaling; #9101S: Certificate of analysis states "This document certifies that this product has met all of the quality control standards defined by Cell Signaling Technology, Inc." (https://www.cellsignal.com/products/primary-antibodies/phospho-p44-42-mapk-erk1-2-thr202-tyr204-antibody/9101). Also see: Lu Y, Sareddy GR, Wang J, Wang R, Li Y, Dong Y, Zhang Q, Liu J, O'Connor JC, Xu J, Vadlamudi RK, Brann DW. Neuron-Derived Estrogen Regulates Synaptic Plasticity and Memory. J Neurosci. 2019 Apr 10;39(15):2792-2809. doi: 10.1523/JNEUROSCI.1970-18.2019. Epub 2019 Feb 6. PMID: 30728170; PMCID: PMC6462452.

α-EpCAM; Cell Signaling; #93790S: Certificate of analysis states "This document certifies that this product has met all of the quality

control standards defined by Cell Signaling Technology, Inc." (https://media.cellsignal.com/coa/93790/1/93790-lot-1-coa.pdf).

α-Occludin; Invitrogen; 71-1500; Cited in 328 publications to date and is validated by Thermo Fisher. Also see: Alvarez, J. I., Dodelet-Devillers, A., Kebir, H., Ifergan, I., Fabre, P. J., Terouz, S., . . . Prat, A. (2011). The Hedgehog PATHWAY Promotes blood-brain Barrier integrity and CNS IMMUNE QUIESCENCE. Science, 334(6063), 1727-1731. doi:10.1126/science.1206936

α-CD3; Cell Signaling; #78588S: Certificate of analysis states "This document certifies that this product has met all of the quality control standards defined by Cell Signaling Technology, Inc." (https://media.cellsignal.com/coa/78588/1/78588-lot-1-coa.pdf). Functionality was confirmed by manufacturer with confocal immunofluorescent analysis of mouse spleen and pancreas.

List of primary and secondary antibodies and their validation for immunoblotting on mouse tissues. The list includes information provided by the manufactures as well as relevant publications for each antibody, application, species specificity.

Primary Antibodies; SOURCE; IDENTIFIER
α-KRAS; Abcam; ab180772: Antibody is validated for use with mouse by Abcam (https://www.abcam.com/kras-antibody-ab180772.html). Experimental down-regulation of KRAS in Figure 6D of this manuscript further validates KRAS reactivity of the antibody. Also see: He F et al. Reversal of EGFR inhibitors' resistance by co-delivering EGFR and integrin avß3 inhibitors with nanoparticles in non-small cell lung cancer. Biosci Rep 39:N/A (2019).

α-pERK1/2; Cell Signaling; #9101S: Certificate of analysis states "This document certifies that this product has met all of the quality control standards defined by Cell Signaling Technology, Inc." (https://www.cellsignal.com/products/primary-antibodies/phospho-p44-42-mapk-erk1-2-thr202-tyr204-antibody/9101). Also see: Gorinski N, Bijata M, Prasad S, et al. Attenuated palmitoylation of serotonin receptor 5-HT1A affects receptor function and contributes to depression-like behaviors. Nat Commun. 2019;10(1):3924. Published 2019 Sep 2. doi:10.1038/s41467-019-11876-5

α-ERK1/2; BD Transduction Laboratories; #610123: Antibody was tested in development by manufacturer and quality control is performed on mouse samples by the manufacturer (https://www.bdbiosciences.com/us/applications/research/stem-cell-research/stem-cell-signaling/human/purified-mouse-anti-erk-pan-erk-16erk-pan-erk/p/610123). Also see: Rajbhandari, N.; W.C. Lin; B.L. Wehde, A.A. Triplett and K.U. Wagner (2017): Autocrine IGF1 signaling mediates pancreatic tumor cell dormancy in the absence of oncogenic drivers.Cell Rep. 18 (9): 2243-2255.

α-GAPDH; Cell Signaling; #5174S: Certificate of analysis states "This document certifies that this product has met all of the quality control standards defined by Cell Signaling Technology, Inc." (https://www.cellsignal.com/products/primary-antibodies/gapdh-d16h11-xp-rabbit-mab/5174). Also see: Wehde BL, Rädler PD, Shrestha H, Johnson SJ, Triplett AA, Wagner KU. Janus Kinase 1 Plays a Critical Role in Mammary Cancer Progression. Cell Rep. 2018;25(8):2192-2207.e5. doi:10.1016/j.celrep.2018.10.063

α-E-cadherin; Cell Signaling; #3195: Certificate of analysis states "This document certifies that this product has met all of the quality control standards defined by Cell Signaling Technology, Inc." (https://www.cellsignal.com/products/primary-antibodies/e-cadherin-24e10-rabbit-mab/3195). Also see: Wu Y, Chen K, Xing G, et al. Phospholipid remodeling is critical for stem cell pluripotency by facilitating mesenchymal-to-epithelial transition. Sci Adv. 2019;5(11):eaax7525. Published 2019 Nov 27. doi:10.1126/sciadv.aax7525

α-N-cadherin; Cell Signaling; #14215: Certificate of analysis states "This document certifies that this product has met all of the quality control standards defined by Cell Signaling Technology, Inc." (https://www.cellsignal.com/products/primary-antibodies/n-cadherin-13a9-mouse-mab/14215?site-search-type=Products&N=4294956287&Ntt=%2314215&fromPage=plp&_requestid=512665). Also see: Liu C, Wang J, Zhao L, et al. Knockdown of Thymidine Kinase 1 Suppresses Cell Proliferation, Invasion, Migration, and Epithelial-Mesenchymal Transition in Thyroid Carcinoma Cells. Front Oncol. 2020;9:1475. Published 2020 Jan 29. doi:10.3389/fonc.2019.01475

α-EpCAM; Cell Signaling; #93790S: Certificate of analysis states "This document certifies that this product has met all of the quality control standards defined by Cell Signaling Technology, Inc." (https://www.cellsignal.com/products/primary-antibodies/epcam-e6v8y-xp-rabbit-mab-mouse-preferred/93790). Validated in multiple mouse tissues by Cell Signaling.

α-CK14; Covance; PRB-155P: "Each lot of this antibody is quality control tested by immunohistochemical staining" (https://www.biolegend.com/en-us/products/keratin-14-polyclonal-antibody-purified-10953)

α-cleaved Caspase 3; Cell Signaling; #9661S: Certificate of analysis states "This document certifies that this product has met all of the quality control standards defined by Cell Signaling Technology, Inc."(https://www.cellsignal.com/products/primary-antibodies/cleaved-caspase-3-asp175-antibody/9661). Also see: Rajbhandari, N.; W.C. Lin; B.L. Wehde, A.A. Triplett and K.U. Wagner (2017): Autocrine IGF1 signaling mediates pancreatic tumor cell dormancy in the absence of oncogenic drivers.Cell Rep. 18 (9): 2243-2255.

α-ITGB3 (CD61); Abcam; ab75872; Validated by Abcam (https://www.citeab.com/antibodies/767994-ab75872-anti-integrin-beta-3-antibody-epr2417y) Also see: Sun SJ, Wu CC, Sheu GT, et al. Integrin β3 and CD44 levels determine the effects of the OPN-a splicing variant on lung cancer cell growth. Oncotarget. 2016;7(34):55572-55584. doi:10.18632/oncotarget.10865).

α-GATA3; Cell Signaling; #5852T: Certificate of analysis states "This document certifies that this product has met all of the quality control standards defined by Cell Signaling Technology, Inc." (https://www.cellsignal.com/products/primary-antibodies/gata-3-d13c9-xp-rabbit-mab/5852). Also see: Ghonim MA, Wang J, Ibba SV, et al. Sulfated non-anticoagulant heparin blocks Th2-induced asthma by modulating the IL-4/signal transducer and activator of transcription 6/Janus kinase 1 pathway. J Transl Med. 2018;16(1):243. Published 2018 Sep 1. doi:10.1186/s12967-018-1621-5.

α-SLUG; Cell Signaling; #9585T: Certificate of analysis states "This document certifies that this product has met all of the quality control standards defined by Cell Signaling Technology, Inc." (https://www.cellsignal.com/products/primary-antibodies/slug-c19g7-rabbit-mab/9585). Also see: Yang, Y., Bae, W.K., Lee, J. et al. Potassium usnate, a water-soluble usnic acid salt, shows enhanced bioavailability and inhibits invasion and metastasis in colorectal cancer. Sci Rep 8, 16234 (2018). https://doi.org/10.1038/s41598-018-34709-9

α-SNAIL; Cell Signaling; #3879T: Certificate of analysis states "This document certifies that this product has met all of the quality control standards defined by Cell Signaling Technology, Inc." (https://www.cellsignal.com/products/primary-antibodies/snail-c15d3-rabbit-mab/3879). Also see: Yang, Y., Bae, W.K., Lee, J. et al. Potassium usnate, a water-soluble usnic acid salt, shows enhanced bioavailability and inhibits invasion and metastasis in colorectal cancer. Sci Rep 8, 16234 (2018). https://doi.org/10.1038/s41598-018-34709-9

α-ERBB2; Calbiochem; OP-15: The manufacturer states that this antibody is "validated for WB, IF, IP, and IHC on frozen and paraffin sections" (https://www.emdmillipore.com/US/en/product/Anti-c-ErbB2-c-Neu-Ab-3-Mouse-mAb-3B5,EMD_BIO-OP15#documentation). Also see: Váradi T, Mersich T, Auvinen P, et al. Binding of trastuzumab to ErbB2 is inhibited by a high pericellular density of hyaluronan. J Histochem Cytochem. 2012;60(8):567-575. doi:10.1369/0022155412448070

α-p53; Cell Signaling; #2524T: Certificate of analysis states "This document certifies that this product has met all of the quality control standards defined by Cell Signaling Technology, Inc." (https://www.cellsignal.com/products/primary-antibodies/p53-1c12-mouse-mab/2524). Also see: Benkafadar N, François F, Affortit C, et al. ROS-Induced Activation of DNA Damage Responses Drives Senescence-Like State in Postmitotic Cochlear Cells: Implication for Hearing Preservation. Mol Neurobiol. 2019;56(8):5950-5969. doi:10.1007/s12035-019-1493-6

α-MDM2; Oncogene; OP115T-10UG. Validated on mouse tissue in Arena G, Cissé MY, Pyrdziak S, et al. Mitochondrial MDM2 Regulates Respiratory Complex I Activity Independently of p53. Mol Cell. 2018;69(4):594-609.e8. doi:10.1016/j.molcel.2018.01.023

α-p19Arf/p16Ink4a; Abcam; ab80: The manufacturer confirms that the antibody "detects a specific band in WT mouse embryo fibroblasts which is not present in p19ARF-null MEFs." (https://www.abcam.com/cdkn2ap19arf-antibody-ab80.html). See also: Seoane M, Costoya JA, Arce VM. Uncoupling Oncogene-Induced Senescence (OIS) and DNA Damage Response (DDR) triggered by DNA hyper-replication: lessons from primary mouse embryo astrocytes (MEA). Sci Rep. 2017;7(1):12991. Published 2017 Oct 11. doi:10.1038/s41598-017-13408-x

α-ER; Abcam; ab32063: Validated by Abcam (https://www.abcam.com/estrogen-receptor-alpha-antibody-e115-chip-grade-ab32063.html). See also: Sarkar S, Ghosh A, Banerjee S, Maity G, Das A, Larson MA, Gupta V, Haque I, Tawfik O, Banerjee SK. CCN5/WISP-2 restores ER-∝ in normal and neoplastic breast cells and sensitizes triple negative breast cancer cells to tamoxifen. Oncogenesis. 2017 May 22;6(5):e340. doi: 10.1038/oncsis.2017.43. PMID: 28530705; PMCID: PMC5569333.

α-PR; Abcam; ab2765: Validated by Abcam (https://www.abcam.com/progesterone-receptor-antibody-alpha-pr6-ab2765.html). Also see: Li R, Wu J, He J, Wang Y, Liu X, Chen X, Tong C, Ding Y, Su Y, Chen W, Zhang C, Gao R. Mice endometrium receptivity in early pregnancy is impaired by maternal hyperinsulinemia. Mol Med Rep. 2017 May;15(5):2503-2510. doi: 10.3892/mmr.2017.6322. Epub 2017 Mar 14. PMID: 28447735; PMCID: PMC5428841.

α-TWIST; Santa Cruz; sc81417: Validated by Santa Cruz (https://www.scbt.com/p/twist-antibody-twist2c1a). Also see: Tripolt S, Neubauer HA, Knab VM, Elmer DP, Aberger F, Moriggl R, Fux DA. Opioids drive breast cancer metastasis through the δ-opioid receptor and oncogenic STAT3. Neoplasia. 2021 Feb;23(2):270-279. doi: 10.1016/j.neo.2020.12.011. Epub 2021 Jan 16. PMID: 33465556; PMCID: PMC7815495.

α-ZEB1; Cell Signaling; #70512T: Certificate of analysis states "This document certifies that this product has met all of the quality control standards defined by Cell Signaling Technology, Inc." (https://media.cellsignal.com/coa/70512/1/70512-lot-1-coa.pdf). Validated by Cell Signaling (https://www.cellsignal.com/products/primary-antibodies/zeb1-e2g6y-xp-rabbit-mab/70512).

Eukaryotic cell lines

Policy information about cell lines

Cell line source(s)	HC11 (ATCC, CRL-3062) and NIH3T3 (CRL-1658) cells were used in selected experiments as controls. As an additional control, we used p53-deficient mouse embryonic fibroblasts (MEFs) generated in our laboratory (PMC1201394). For this study, we generated three new mammary tumor cell lines expressing oncogenic KRAS.
Authentication	The correct genotype of the mammary tumor cell lines was validated by PCR using transgene-specific primer sets (WAP-Cre, EF1-LSL-tTA, TetO-Kras, and CAG-LSL-GFP). The genotypes of the cell lines corresponded precisely to the genotypes of the mice from which they were derived. Moreover, the presence of GFP expression in all cell lines was indicative that we derived pure cancer cell lines, and the Dox-mediated downregulation of the oncogene (KRAS) was yet another validation for the correct genotype.
Mycoplasma contamination	Mycoplasma contamination was not detected in the primary cells or the the cell lines acquired from ATCC. The parental mice that were used to derive the cell lines were pathogen-free as indicated in the sentinel reports.
Commonly misidentified lines (See ICLAC register)	No commonly misidentify lines were used in this study for experiments.

Animals and other organisms

Policy information about studies involving animals; ARRIVE guidelines recommended for reporting animal research

Laboratory animals	Species: Mus musculus; The following genetically engineered lines were used: EF1-LSL-tTA [Eef1a1tm1(tTA)Kuw] and Rosa26CAG-FSF-GFP [Gt(ROSA)26Sortm1.2(CAG-EGFP)Fsh] kockin mice; MMTV-tTA [Tg(MMTV-tTA)25754Kuw], WAP-Cre [Tg(Wap-cre)11738Mam], TetO-KrasG12D [Tg(tetO-Kras2)12Hev/J], TetO-H2B-GFP (Tg(tetO-HIST1H2BJ/GFP)47Efu/J), and CAG-LSL-GFP transgenic mice; FSF-KrasG12D [Krastm5Tyj/J] mutants. These lines were generated in or backcrossed to FVB/N genetic background as described in the manuscript. Athymic nude mice (NCr nu/nu females) were used for transplantation of mouse tumor cells.<br>All mice were housed under pathogen-free conditions in micro-isolator cages in an environmentally controlled room (22 °C, with 50 ± 10% humidity) on a 12/12-hour light/dark cycle.
Wild animals	The study did not involve wild animals.
Field-collected samples	The study did not involve samples collected from the field.
Ethics oversight	This work was conducted in accordance with the recommendations in the Guide for the Care and Use of Laboratory Animals of the National Institutes of Health. The animal study protocols were approved by the Institutional Animal Care and Use Committee (IACUC) of the Nebraska Medical Center and Wayne State University (18-02-0538, 19-07-1196, 19-07-1190).

Note that full information on the approval of the study protocol must also be provided in the manuscript.

Flow Cytometry

Plots

Confirm that:

- [x] The axis labels state the marker and fluorochrome used (e.g. CD4-FITC).
- [x] The axis scales are clearly visible. Include numbers along axes only for bottom left plot of group (a 'group' is an analysis of identical markers).
- [x] All plots are contour plots with outliers or pseudocolor plots.
- [x] A numerical value for number of cells or percentage (with statistics) is provided.

Methodology

Sample preparation	The flow cytometric analysis for the expression of mammary cell markers (CD61, CD24, CD29, CD49f) was carried out by incubating the cells with primary antibodies against the respective antigen in FACS buffer (1x PBS supplemented with 2% bovine serum albumin) for 25 min at 4°C. Subsequently, cells were washed once in ice-cold FACS buffer to remove excess antibodies. PE, PE/Cy7, and APC-conjugated monoclonal antibodies against CD24 (BD Pharmingen, 553262, 1:70 dilution), CD49f (BioLegend, 313621, 1:40 dilution), and CD61 (Thermo Fisher Scientific, MCD6105, 1:100 dilution) were used.
Instrument	BD Biosciences LSRII
Software	FACSdiva 8.0.1 software
Cell population abundance	All single stained cells were of interest. The cells displayed in the graphs are single cells (aggregates and dead cells were excluded). >85% of cells were singlets and >95% of the singlets were alive.
Gating strategy	First, sample quality was assessed via SSC-A vs Time and any bubbles or areas of stream instability were removed prior to analysis. Cells were gated using FSC-A vs SSC-A. Aggregates were excluded by doublet discrimination using FSC-A vs. FSC-H. Dead cells were excluded using FSC-A vs. the DAPI viability dye.

- [x] Tick this box to confirm that a figure exemplifying the gating strategy is provided in the Supplementary Information.
